# The Interplay Among Perceived Teacher-Student Rapport, Academic Engagement, and L2 Grit: A Structural Equation Modelling Approach

**DOI:** 10.11621/pir.2025.0302

**Published:** 2025-09-01

**Authors:** Mostafa Janebi Enayat, Snur Xudaie

**Affiliations:** a University of Maragheh, Iran

**Keywords:** perceived teacher-student rapport, academic engagement, L2 grit, EFL context, structural equation modelling

## Abstract

**Background:**

The positive role of grit (perseverance and passion for long-term goals even in the face of setbacks) in language learning outcomes is well-documented in the academic literature. However, a significant gap remains in understanding the factors that contribute to the development and enhancement of second language (L2) grit.

**Objective:**

The present study explored the interplay among perceived teacher-student rapport, academic engagement, and L2 grit from the perspective of Iranian EFL learners. More specifically, the predictive role of teacher-student rapport and academic engagement in Iranian EFL learners’ grit was probed. Additionally, the mediating role of academic engagement in the relationship between teacher-student rapport and L2 grit was examined.

**Design:**

To this end, 397 Iranian undergraduate and graduate students were selected using snowball sampling from different universities in Iran. Structural equation modelling (SEM) was used to analyse the data.

**Results:**

Both teacher-student rapport and academic engagement were significant predictors of L2 grit, with engagement identified as the strongest predictor. Moreover, academic engagement served as a mediating variable in the relationship between teacher-student rapport and L2 grit, underscoring its pivotal role in cultivating learners’ perseverance and passion for second language acquisition.

**Conclusion:**

The findings suggest that positive emotions and supportive social interactions enhance cognitive and behavioural capacities, thereby fostering a more resilient approach to learning. Relevant implications and suggestions for future research are discussed.

## Introduction

Positive psychology is grounded in the *broaden-and-build theory* of positive emotions, which posits that emotions such as joy, love, and interest “*broaden* people’s momentary thought-action repertoires and *build* their enduring personal resources” (Fredrickson, 2004, p. 1369). Positive emotions are crucial predictors of success in L2 learning, as language teaching is inherently emotional (Dewaele et al., 2019; Wang et al., 2021). Language educators play a pivotal role in fostering these emotions, enriching learners’ perspectives, and facilitating deeper engagement with the language (Mystkowska-Wiertelak, 2020).

The *broaden-and-build theory* (Fredrickson, 2001) suggests that positive emotions, which are often fostered through strong teacher-student rapport, broaden learners’ cognitive and behavioural repertoires, thereby enhancing their engagement and resilience. Learners with higher grit levels are better prepared to overcome the challenges of language learning, such as expanding their vocabulary size and depth (El Hadim & Ghaicha, 2024) and enhancing critical skills like writing (Zhang, 2023; Zhang & Zhang, 2023) and speaking (Sun et al., 2024). Despite the increasing acknowledgment of grit’s influence on language learning outcomes, there remains a significant gap in understanding the factors that contribute to the development and enhancement of second language (L2) grit.

Teacher-student rapport refers to the quality of interpersonal relationships between educators and learners, characterised by mutual respect, trust, and understanding (Frisby & Martin, 2010). Within the context of language learning, Mercer and Dörnyei (2020) highlight rapport as a relational bond that builds an environment where students feel appreciated, respected, and engaged to actively participate in learning activities. Consequently, teacher-student rapport not only cultivates a positive classroom atmosphere but also establishes a relational foundation that enhances student engagement and emotional well-being. Empirical research demonstrates the beneficial impact of teacher-student rapport on various academic and psychological outcomes, including second language (L2) achievement and learner engagement. For example, Wilson et al. (2010) developed a teacher-student rapport scale and found it to be a significant predictor of outcomes such as student motivation, perceived learning, and self-reported academic performance. Similarly, van Uden et al. (2014) explored the relationship between perceived teacher-student interactions and specific teacher beliefs, identifying interpersonal teacher behaviour as the strongest predictor of diverse forms of student engagement. More recent studies have further corroborated these findings, revealing substantial associations between teacher-student rapport and factors such as willingness to communicate (WTC) (*e.g*., Zhi & Wang, 2024), academic motivation (*e.g*., Derakhshan et al., 2025; Pourgharib & Shakki, 2024), and academic engagement (*e.g.*, Derakhshan et al., 2022; Li, 2024; Shakki, 2022; Yuan, 2024).

Another key variable within the area of positive psychology is engagement, which is broadly defined as the extent to which learners exhibit interest and actively participate in their learning processes (Hiver et al., 2021). Schaufeli et al. (2002) conceptualise academic engagement through three key dimensions: vigour, dedication, and absorption. Vigour denotes a state of heightened energy and mental resilience, enabling students to actively engage and persist even when facing challenges. Dedication encompasses a sense of purpose, aspiration, and commitment to one’s studies, often driving intrinsic motivation (Fredricks et al., 2016). Absorption denotes deep, focussed involvement in academic tasks, where students become so immersed that they lose track of time. Research has demonstrated that highly engaged language learners achieve greater linguistic proficiency, heightened motivation, and improved adaptation to complex language tasks (Al-Obaydi et al., 2023; Hiver et al., 2021). For instance, Hao and Lu (2024) explored the interplay between self-efficacy and engagement in EFL writing, finding that self-efficacy positively influenced writing achievement, with engagement serving as a mediating factor. Similarly, Namaziandost et al. (2024) investigated how learner autonomy and academic engagement predict Iranian EFL learners’ WTC, self-esteem, and L2 grit. Their findings demonstrated that both autonomy and engagement were significant predictors, highlighting their essential role in facilitating successful language acquisition. The interrelationship between teacher-student rapport and engagement was also established by Shakki (2022).

The final variable of this study is L2 grit, which was derived from Duckworth et al.’s (2007) broader concept of grit and is defined as a learner’s perseverance and sustained interest in mastering a second language despite challenges. This construct consists of two primary dimensions: *perseverance of effort*, which reflects consistent effort and resilience in the face of obstacles, and *consistency of interest*, which pertains to maintaining passion and focus on language learning goals over time (Duckworth et al., 2007; Teimouri et al., 2022). The significance of L2 grit lies in its impact on learner resilience and success within second language acquisition (SLA). Recent studies have highlighted the pivotal role of L2 grit in enhancing language learning outcomes. Teimouri et al. (2022) demonstrated that learners with higher grit levels achieved greater L2 proficiency and motivation. In another study, El Hadim and Ghaicha (2024) investigated the link between L2 grit and vocabulary acquisition among Moroccan EFL learners, revealing that both perseverance of effort and consistency of interest significantly contributed to vocabulary knowledge in undergraduate students. Similarly, Zhang and Zhang (2023) and Zhang (2023) provided evidence of a strong positive relationship between L2 grit and EFL writing performance. The dynamic interaction between L2 grit, motivational intensity, WTC, and perceived speaking performance was examined by Sun et al. (2024). Their findings indicated that while both dimensions of L2 grit influenced motivational intensity, their impact on speaking ability was mediated through WTC and learner motivation, further emphasising the multifaceted role of L2 grit in language learning. In a more recent study, Bensalem et al. (2025) examined the contribution of grit to the prediction of EFL learners’ WTC in a blended learning environment and found grit as an influential variable. The dynamic interaction between grit and academic engagement has been reported by Derakhshan and Fathi (2024). Additionally, L2 grit has been reported as a predictor variable of boredom coping strategies, which could result in higher levels of engagement (Solhi et al., 2023).

Within the framework of positive psychology, it is argued that learners who benefit from supportive and positive relationships are more inclined to develop the perseverance required for sustained academic success (Dörnyei & Ryan, 2015). Consequently, engagement may serve as a mediating factor linking the supportive learning environment created by teacher-student rapport to learners’ long-term perseverance. Despite the recognised importance of these constructs, the interplay between teacher-student rapport, academic engagement, and L2 grit remains underexplored in SLA research. While existing studies have independently associated teacher-student rapport and academic engagement with positive language learning outcomes, limited attention has been given to how these factors interact collectively to shape learners’ L2 grit. Accordingly, this study aims to address the following research questions:

1) Do perceived teacher-student rapport and academic engagement predict L2 grit?
2) Can academic engagement mediate the relationship between teacher-student rapport and L2 grit?

## Method

### Participants

A sample of 397 undergraduate and graduate students, recruited through snowball sampling, participated in the present study. The participants age ranged from 18 to 36 (*M* = 22.48, *SD* = 2.85). The demographic information of the participants provided in *Table 1* shows that most of the participants were female undergraduate students, and the major of 173 students was English Language Teaching.

**Table 1:** Demographic Information of the Participants

Categories	Gender	N	Percent
Gender	Female	260	65.49
	Male	137	34.51
Education	Bachelor’s degree	257	64.74
	Master’s degree	109	27.46
	Ph.D.	25	6.29
	Other	6	1.10
Major	English Language and Literature	126	31.73
	English Language Teaching	173	43.56
	English Language Translation	98	24.68

### Instruments

#### Utrecht Work Engagement Scale for Students (UWES-S)

The UWES-14S (Schaufeli et al., 2002) is a 14-item instrument designed to assess the students’ perceived academic engagement across three subscales: vigour (five items), dedication (five items), and absorption (four items). Responses are rated on a seven-point Likert scale, ranging from 0 (never) to 6 (always). Derakhshan et al. (2022) reported high reliability for the scale, with Cronbach’s alpha coefficients of α = .92 for the overall engagement scale and α = .84, α = .84, and α = .91 for the vigour, dedication, and absorption subscales, respectively. The construct validity of the scale was also verified for the Iranian population by Derakhshan et al. (2022).

#### Professor-Student Rapport Scale

Teacher-student rapport was examined by a scale which was designed by Wilson et al. (2010). This one-dimensional, 34-item instrument evaluates the perceived rapport between students and one of their teachers. The items are rated on a five-point Likert scale, with responses ranging from 1 (strongly disagree) to 5 (strongly agree). The scale demonstrates excellent reliability, with Wilson et al. (2010) reporting a Cronbach’s alpha coefficient of .96. Similarly, Derakhshan et al. (2022) confirmed its high reliability in their study, reporting a Cronbach’s alpha coefficient of .95. Moreover, the authors verified the construct validity of this scale for the Iranian population.

#### L2 Grit Scale

To assess the participants’ L2 grit, the present study employed the scale designed by Teimouri et al. (2022), which encompasses two interconnected subconstructs: Perseverance of Effort (POE) and Consistency of Interest (COI). The nine-item instrument is based on the theoretical framework of grit outlined by Duckworth et al. (2007, 2016) and is assessed using a five-point Likert scale, with responses ranging from 1 (not at all like me) to 5 (very much like me). Teimouri et al. (2022) reported a Cronbach’s alpha coefficient of .75 for the overall scale, with reliability estimates of α = .75 for POE and α = .78 for COI, indicating satisfactory internal consistency. The scale has been validated for the Iranian population in a few studies (*e.g.*, Elahi Shirvan et al., 2021; Khajavy et al., 2021).

### Data Collection

An online survey was developed using Google Forms, which included the three scales used in this study. The survey link was distributed to participants through social network like Telegram and WhatsApp to ensure wide accessibility. Informed consent was obtained prior to participation, and participants were assured that their responses would remain confidential. Since all participants were enrolled in English-related majors, the survey was administered in English. For the teacher-student rapport scale, respondents were instructed to focus on a single EFL instructor when completing all relevant items, ensuring consistency in their responses. However, this was not followed for the other two scales as they did not measure relationships with a specific teacher.

### Data Analysis

The dataset was first examined using SPSS version 27.0 to detect any outliers or missing data, confirming the absence of such issues. Following this, the necessary assumptions for performing confirmatory factor analysis (CFA) and structural equation modeling (SEM) in AMOS version 26.0 were checked. The data distribution was found to be normal, with skewness and kurtosis values falling within Kunnan’s (1998) recommended range of -2.0 to +2.. Multicollinearity was assessed, and variance inflation factor (VIF) values were all below the recommended threshold of 5 (Larson-Hall, 2015). Next, CFA was conducted to evaluate the validity of the measurement scales used in this study.

The initial dataset, which included responses from 401 participants, underwent cleaning to remove any problematic cases. All data were accounted for, but four responses showing irregular patterns, with constant, increasing, or decreasing trends were removed from the analysis. After removing these responses, the final sample consisted of 397 valid responses for the three scales used in the study. The standard deviation of responses per participant exceeded the threshold of .5, ensuring that there were no disengaged responses in the final dataset.

## Results

### Validity and Reliability of the Scales

**Figure 1.**
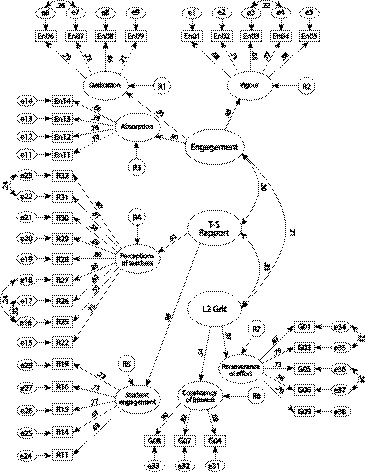
The Modified CFA Model with Standardised Estimates

To ensure the validity and reliability of the scales used in the study, CFA was conducted. *Table 2* presents both the unstandardised and standardised estimates derived from the analysis. The results indicated that all the items displayed significant loadings on their respective factors. However, item 10 from the academic engagement scale, item 17 from the teacher-student rapport scale, and item 2 from the L2 grit scale had standardised loadings below .5. According to Kline (2016), such low loadings may threaten the model’s convergent validity. Consequently, these items were excluded from further analysis. Moreover, the modification indices were inspected to spot the shared errors that could improve the model fit. The items whose content suggested that they could have shared errors and connecting their errors improved the model by at least 10 parameter loading were taken into account. *Figure 1* displays the final model of CFA, including the standardised estimates.

**Table 2:** Unstandardised and Standardised Estimates of the First CFA Model

			Unstandardised				Standardised
			Estimate	S.E.	C.R.	p	Estimate
Dedication	<---	Academic Engagement	1.000				.900
Vigour	<---	Academic Engagement	.879	.069	12.779	.000	.968
Absorption	<---	Academic Engagement	.954	.076	12.562	.000	.900
Consistency of Interest	<---	L2 Grit	1.000				.549
Perseverance of Effort	<---	L2 Grit	1.429	.183	7.823	.000	.970
Perceptions of Teachers	<---	Rapport	1.000				.910
Student Engagement	<---	Rapport	.965	.082	11.805	.000	.981
En01	<---	Vigour	1.000				.676
En02	<---	Vigour	1.195	.094	12.715	.000	.715
En03	<---	Vigour	1.103	.090	12.196	.000	.682
En04	<---	Vigour	1.236	.089	13.945	.000	.796
En05	<---	Vigour	1.114	.105	1.592	.000	.584
En06	<---	Dedication	1.000				.820
En07	<---	Dedication	1.009	.055	18.476	.000	.814
En08	<---	Dedication	1.059	.054	19.668	.000	.852
En09	<---	Dedication	.862	.057	15.190	.000	.703
En10	<---	Dedication	.439	.063	6.968	.000	.356
En11	<---	Absorption	1.000				.697
En12	<---	Absorption	1.147	.085	13.532	.000	.760
En13	<---	Absorption	1.110	.080	13.844	.000	.780
En14	<---	Absorption	.876	.073	11.925	.000	.661
R11	<---	Student Engagement	1.000				.675
R14	<---	Student Engagement	.992	.088	11.249	.000	.629
R15	<---	Student Engagement	1.169	.088	13.271	.000	.760
R16	<---	Student Engagement	1.220	.097	12.592	.000	.715
R17	<---	Student Engagement	.727	.090	8.070	.000	.440
R19	<---	Student Engagement	1.198	.096	12.526	.000	.710
R22	<---	Perceptions of Teachers	1.000				.748
R25	<---	Perceptions of Teachers	.597	.051	11.695	.000	.587
R26	<---	Perceptions of Teachers	.693	.055	12.574	.000	.628
R27	<---	Perceptions of Teachers	1.149	.065	17.587	.000	.850
R28	<---	Perceptions of Teachers	.982	.060	16.275	.000	.794
R29	<---	Perceptions of Teachers	1.005	.073	13.705	.000	.680
R30	<---	Perceptions of Teachers	.841	.074	11.420	.000	.574
R31	<---	Perceptions of Teachers	.975	.057	17.155	.000	.831
R32	<---	Perceptions of Teachers	.999	.059	16.850	.000	.818
G02	<---	Consistency of Interest	1.000				.092
G04	<---	Consistency of Interest	.998	.112	8.906	.000	.695
G07	<---	Consistency of Interest	1.271	.117	1.906	.000	.817
G08	<---	Consistency of Interest	1.415	.126	11.263	.000	.903
G01	<---	Perseverance of Effort	1.000				.677
G03	<---	Perseverance of Effort	1.157	.086	13.392	.000	.763
G05	<---	Perseverance of Effort	1.257	.092	13.623	.000	.778
G06	<---	Perseverance of Effort	1.225	.088	13.971	.000	.802
G09	<---	Perseverance of Effort	1.070	.088	12.187	.000	.684

The CFA model’s fit was assessed using multiple model fit indices. *Table 3* displays the observed values of these indices along with the recommended thresholds for acceptable fit, as outlined by Hu and Bentler (1999). The results indicate that the model exhibited an acceptable to excellent fit, with all indices meeting or exceeding the suggested criteria.

**Table 3:** Evaluation of the CFA Goodness of Fit

Fit Index	Observed Value	Poor	Acceptable	Excellent	Result
CMIN	1051.4				
DF	545				
CMIN/DF	1.929		> 5	> 3	Excellent
RMSEA	.048	> .10	> .08	< .08	Excellent
CFI	.936	< .9	> .9	> .95	Acceptable
TLI	.930	< .9	> .9	> .95	Acceptable
SRMR	.06	> .10	> .08	< .08	Excellent

*Note. CMIN = Chi-square; DF = degrees of freedom; RMSEA = Root Mean Square Error of Approximation; CFI = Comparative Fit Index; TLI = Tucker-Lewis Index; SRMR = Standardised Root Mean Square Residual.*

The reliability and construct validity of the model were further evaluated (*Table 4*). Composite Reliability (CR) values, which reflect the internal consistency of each construct, exceeded the recommended threshold of .7, confirming their reliability. The Average Variance Extracted (AVE), a measure of the proportion of variance captured by each construct and an indicator of convergent validity, was also assessed. The AVE for academic engagement was .869, indicating a high proportion of variance explained by the construct and supporting its validity. For teacher-student rapport, the AVE was .897, while L2 grit had an AVE of .617, both of which meet the acceptable threshold of .5.

**Table 4:** Reliability and Validity of the Variables

					Fornell – Larcker Criterion		
	CR	AVE	MSV	MaxR(H)	Engagement	Rapport	L2 Grit
Academic Engagement	.952	.869	.590	.975	.932		
Teacher-Student Rapport	.946	.897	.110	.968	.296**	.947	
L2 Grit	.749	.617	.590	.940	.719**	.332**	.786

Note.** Correlation Signiftcant at p < .01

The Maximum Shared Variance (MSV), an indicator of discriminant validity, revealed a highest MSV value of .59, which is significantly lower than the AVE values for the corresponding constructs, confirming sufficient discriminant validity. Additionally, Maximum Reliability (MaxR(H)) was calculated, with all values meeting the recommended thresholds, further supporting the model’s reliability. These criteria were established by Hu and Bentler (1999).

The Fornell-Larcker criterion also approved the model’s discriminant validity. According to Fornell and Larcker (1981), discriminant validity is achieved when the square root of the AVE for each construct surpasses its correlations with other constructs. As shown in *Table 4*, this condition was met for all three constructs.

The correlations among the three variables are also presented in *Table 4.* As reported, there was a strong and significant positive correlation between academic engagement and L2 grit (*r* = .719, *p* < .01). Moreover, teacher-student rapport exhibited moderate and significant correlations with both academic engagement (*r* = .296, *p* < .01) and L2 grit (*r* = .332, *p* < .01).

### The Research Questions

**Figure 2.**
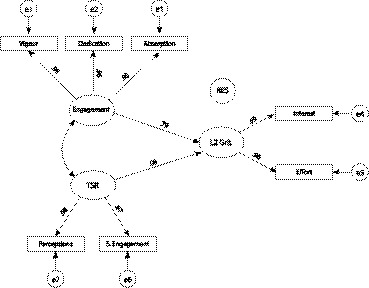
The Measurement Model with Standardised Estimates

A regression-based measurement model was created to answer the first research question. First, the components and constructs were estimated through regression imputation, and, subsequently, the measurement model was developed. *Table 5* presents the outcomes derived from this model, and *Figure 2* illustrates the model with standardised estimates. The structural equation modelling (SEM) analysis revealed that teacher-student rapport and academic engagement collectively accounted for 74.9% of the variance in L2 grit. Although both variables were statistically significant predictors of L2 grit, academic engagement exhibited a notably higher beta coefficient (.79) compared to teacher-student rapport (.09). Consequently, a mediation analysis was conducted to determine whether academic engagement mediated the relationship between teacher-student rapport and L2 grit (RQ2). The results (Indirect effect = .188, p = .007, 95% CI [.186, .191]) confirmed that academic engagement served as a significant mediator within the model. The measurement model demonstrated acceptable to excellent fit indices: χ^2^/df = 3.57, RMSEA = .071, CFI = .984, TLI = .973, and SRMR = .045.

**Table 5:** Results of Regression from SEM

			Unstandardised				β	R^2^ Multiple Correlation
			Estimate	S.E.	C.R.	P		
L2 Grit	<---	Academic Engagement	.470	.035	13.334	.000	.793	.749
L2 Grit	<---	Teacher-Rapport Student	.061	.019	3.153	.002	.084	
Academic Engagement	<-->	Teacher-Rapport Student	.184	.033	5.557	.000	.297	

## Discussion

The current study aimed to probe the interplay between teacher-student rapport, academic engagement, and grit among Iranian EFL learners. The results indicated that rapport and academic engagement were significant predictors of L2 grit, with engagement having the strongest predictive power. Furthermore, academic engagement emerged as a potential mediator in the relationship between rapport and L2 grit, functioning as a mechanism through which these two constructs are interconnected.

The finding that teacher-student rapport is a significant predictor of L2 grit aligns well with the tenets of positive psychology, which emphasise the power of supportive social relationships in enhancing resilience and motivation (Bensalem et al., 2025; Derakhshan & Fathi, 2024; Dewaele & MacIntyre, 2014). Teacher-student rapport — defined by trust, mutual respect, and emotional connection (Martin, 2010) — has been linked to increased motivation and perseverance in language learning contexts. When the teachers are perceived as approachable and understanding, the students are more likely to express their difficulties and seek help when faced with obstacles. This open communication fosters a growth mindset, helping students see difficulties as opportunities for growth rather than insurmountable barriers (Dörnyei & Ushioda, 2011). As such, strong rapport can bolster students’ intrinsic motivation, leading to enhanced effort and commitment toward achieving language learning goals.

The predictive role of teacher-student rapport in L2 grit can be understood through Fredrickson’s *broaden-and-build theory*, which argues that positive emotions and supportive social interactions expand cognitive and behavioural capacities, thereby promoting a more resilient approach to learning (Fredrickson, 2001). Within the EFL classroom, the affective bond between students and teachers can broaden learners’ engagement and instill a sense of purpose, which in turn strengthens their perseverance (Derakhshan et al., 2023). This interplay between rapport and grit suggests that fostering a supportive and engaging environment is a critical component in language learning, as it may facilitate the internal motivation and resilience necessary for long-term achievement (Solhi et al., 2023).

Recent research highlights the link between teacher-student rapport and L2 grit, suggesting that positive teacher-student relationships boost students’ engagement, academic motivation, and commitment to language learning goals (*e.g.*, Pourgharib & Shakki, 2024; Li, 2024; van Uden et al., 2014; Wilson et al., 2010; Yuan, 2024; Zhi & Wang, 2024). These studies emphasise the pivotal role of teacher-student rapport, showing that when students feel respected and supported, they are more likely to develop intrinsic motivation and resilience, which are critical elements for successful language learning (Derakhshan et al., 2023). Moreover, such rapport fosters a psychologically safe learning environment, encouraging learners to take risks, maintain interest, and persevere through challenges in language learning tasks (Shakki, 2022).

The strong predictive effect of academic engagement on L2 grit highlights the critical role that active and sustained involvement in learning plays in building students’ interest and persistence, which are core components of L2 grit (Duckworth et al., 2007). This result is in line with the conceptualisation of engagement as a multidimensional construct, encompassing emotional, cognitive, and behavioural dimensions, each contributing to sustained motivation and effort over time (Schaufeli et al., 2002). The significant relationship between academic engagement and L2 grit aligns with previous studies which reported that highly engaged learners demonstrate improved linguistic skills, heightened motivation, and better adaptation to complex language tasks (*e.g.*, Hao & Lu, 2024; Hiver et al., 2021; Namaziandost et al., 2024). Academic engagement, then, not only directly contributes to language performance but also builds resilience and grit by allowing learners to experience progress and growth in their language studies, even amid challenges (Derakhshan & Fathi, 2024). This suggests that educational practices that cultivate engagement, through interactive tasks, goal-setting, and constructive feedback, could have a significant impact on the development of L2 grit and, consequently, on long-term achievement in language learning.

The mediating role of academic engagement in the relationship between teacher-student rapport and L2 grit offers further insights into the mechanisms by which social and motivational factors influence L2 grit. This finding highlights that academic engagement acts as a bridge connecting external support (*e.g.*, positive teacher-student relationships) with intrinsic motivation factors. When learners experience positive rapport with their teachers, marked by trust, empathy, and a supportive classroom environment, they are more likely to be motivated to participate actively in their studies. This type of rapport can inspire higher levels of academic engagement, as students feel encouraged and valued, contributing to their willingness to commit to language tasks and persevere despite challenges (Derakhshan et al., 2022). Thus, academic engagement not only links rapport to grit but amplifies the impact of positive teacher-student interactions by providing a pathway through which rapport translates into long-term commitment and resilience in L2 learning.

The mediating role of academic engagement aligns with Dörnyei’s (2005) *L2 Motivational Self System*, which suggests that learners’ persistence and success are greatly influenced by their vision of themselves as proficient language users, a vision that engagement helps to sustain. Dörnyei (2005, p. 5) says that “*language learning is a sustained and often tedious process with lots of temporary ups and downs, and I felt that the secret of successful learners was their possession of a superordinate vision that kept them on track*.” He further elaborates that the L2 learning experience, influenced by factors such as the teacher and peer group, constitutes a core component of the L2 Motivational Self System (Dörnyei, 2009). Thus, in line with previous studies (*e.g.*, Bensalem et al., 2025; Derakhshan & Fathi, 2024; Derakhshan et al., 2023), it can be inferred that teacher-student rapport, and academic engagement, work in tandem to create a positive learning environment, which, in turn, enhances students’ L2 grit by fostering motivation, resilience, and sustained effort in language learning.

## Conclusion

This study examined the interplay among teacher-student rapport, academic engagement, and L2 grit in a group of Iranian EFL learners. Results demonstrated that both rapport and academic engagement are key predictors of L2 grit, with academic engagement exerting the most substantial predictive influence. Furthermore, academic engagement emerged as a mediating factor in the rapport-grit relationship, suggesting it functions as a central pathway linking rapport with grit.

The implications of these findings suggest that language educators should prioritise building rapport and promoting active engagement, which, together, contribute to fostering resilient, motivated, and gritty learners. While previous research has linked engagement with achievement outcomes (*e.g.*, Hao & Lu, 2024; Hiver et al., 2021; Namaziandost et al., 2024), this study goes further by highlighting its role as a mediator between rapport and grit, showcasing the intricate dynamics that can reinforce language learners’ resilience in an EFL context. Educators and curriculum designers can leverage these insights to design interventions that not only build rapport but actively engage students, thus helping to cultivate L2 grit and enhance language learning outcomes in diverse educational settings. The results suggest that teachers aiming to foster grit may benefit from adopting engagement-centered strategies, such as providing meaningful feedback, nurturing student autonomy, and incorporating interactive, task-based activities that maintain high levels of interest.

For learners, understanding that engagement can serve as a pathway to developing grit has motivational benefits. When students recognise the role of active engagement in sustaining interest and effort, they may approach language tasks with greater resilience and a long-term commitment to improvement (Teimouri et al., 2022). Furthermore, learner autonomy can be promoted, where students are encouraged to set personal goals, monitor their progress, and reflect on their experiences to deepen their engagement and build grit.

## Limitations

Despite the implications, this study had a few limitations that can be addressed by future research. First, the non-probability sampling technique used for recruiting the EFL learners limit the generalisability of the findings, suggesting that further studies may use more representative samples. Second, the pure quantitative research design of this investigation implies that future studies could obtain more reliable data using qualitative or mixed-methods approaches to research. Finally, other contextual and motivational factors that can contribute to L2 grit could be explored.

## References

[ref01] Al-Obaydi L.H., Shakki F., Tawafak R.M., Pikhart M., & Ugla R.L. (2023). What I know, what I want to know, what I learned: Activating EFL college students’ cognitive, behavioral, and emotional engagement through structured feedback in an online environment. Frontiers in Psychology, 13, 1083673. 10.3389/fpsyg.2022.1083673 36687994 PMC9845881

[ref2] Bensalem E., Derakhshan A., Alenazi F.H., Thompson A.S., & Harizi R. (2025). Modeling the Contribution of Grit, Enjoyment, and Boredom to Predict English as a Foreign Language Students’ Willingness to Communicate in a Blended Learning Environment. Perceptual and Motor Skills, 132(1), 144–168. 10.1177/00315125241289192 39423310

[ref3] Derakhshan A., Dolinski D., Zhaleh K., Janebi Enayat M., & Fathi J. (2022). A mixed-methods cross-cultural study of teacher care and teacher-student rapport in Iranian and Polish University students’ engagement in pursuing academic goals in an L2 context. System, 106, 102790. 10.1016/j.system.2022.102790

[ref4] Derakhshan A.,& Fathi J. (2024). Grit and foreign language enjoyment as predictors of EFL learners’ online engagement: The mediating role of online learning self-efficacy. The Asia-Pacific Education Researcher, 33(4), 759–769. 10.1007/s40299-023-00745-x

[ref5] Derakhshan A., Solhi M., & Azari Noughabi M. (2023). An investigation into the association between student-perceived affective teacher variables and students’ L2-Grit. Journal of Multilingual and Multicultural Development, 46, 798–814. 10.1080/01434632.2023.2212644

[ref6] Derakhshan A., Solhi M., Dewaele J.-M., & Shakki F. (2025). Modeling the associations between L2 teacher support and EFL learners’ reading motivation: The mediating impact of reading enjoyment, anxiety, and boredom. *Studies in Second Language Learning and Teaching,* 1–32. 10.14746/ssllt.40078

[ref7] Dewaele J., Chen X., Padilla A.M., & Lake J. (2019). The flowering of positive psychology in foreign/ second language teaching and acquisition research. Frontiers in Psychology, 10, 2128. 10.3389/fpsyg.2019.02128 31607981 PMC6769100

[ref8] Dewaele J., & MacIntyre P.D. (2014). The two faces of Janus? Anxiety and enjoyment in the foreign language classroom. Studies in Second Language Learning and Teaching, 4(2), 237–274. 10.14746/ssllt.2014.4.2.5

[ref9] Dörnyei Z. (2009). 2. The L2 Motivational self-system. In Dörnyei Z. & Ushioda E. (Eds.), Motivation, language identity and the L2 self (pp. 9–42). Multilingual Matters.

[ref10] Dörnyei Z., & Ryan S. (2015). The psychology of the language learner revisited. Routledge.

[ref11] Dörnyei Z., & Ushioda E. (2011). Motivation, language identity and the L2 self. Multilingual Matters.

[ref12] Duckworth A.L. (2016). Grit: The power of passion and perseverance. Scribner.

[ref13] Duckworth A.L., Peterson C., Matthews M.D., & Kelly D.R. (2007). Grit: perseverance and passion for long-term goals. Journal of Personality and Social Psychology, 92(6), 1087. 10.1037/0022-3514.92.6.1087 17547490

[ref14] Elahi Shirvan M., Taherian T., Shahnama M., & Yazdanmehr E. (2021). A longitudinal study of foreign language enjoyment and L2 grit: A latent growth curve modeling. Frontiers in Psychology, 12, 1–11. 10.3389/fpsyg.2021.720326 PMC8435725 34526939

[ref15] Fornell C., & Larcker D.F. (1981). Evaluating Structural Equation Models with Unobservable Variables and Measurement Error. Journal of Marketing Research, 18(1), 39. 10.2307/3151312

[ref16] Fredricks J.A., Filsecker M., & Lawson M.A. (2016). Student engagement, context, and adjustment: Addressing definitional, measurement, and methodological issues. Learning and Instruction, 43, 1–4. 10.1016/j.learninstruc.2016.02.002

[ref17] Fredrickson B.L. (2001). The role of positive emotions in positive psychology: The broaden-and-build theory of positive emotions. American Psychologist, 56(3), 218–226. 10.1037/0003-066X.56.3.218 11315248 PMC3122271

[ref18] Fredrickson B.L. (2004). The broaden-and-build theory of positive emotions. The Royal Society, 359, 1367–1377. 10.1098/rstb.2004.1512 PMC1693418 15347528

[ref19] Frisby B.N., & Martin M.M. (2010). Instructor-student and student-student rapport in the classroom. Communication Education, 59(2), 146–164. 10.1080/03634520903564362

[ref20] Hadim H.E., & Ghaicha A. (2024). The relationship between L2 grit and vocabulary knowledge in first-year Moroccan university students. System, 123, 103316. 10.1016/j.system.2024.103316

[ref21] Hao X., & Lu H. (2024). The role of self-efficacy and learner engagement in the relationship between prior achievement and final achievement in EFL writing. Learning and Motivation, 87, 102007. 10.1016/j.lmot.2024.102007

[ref22] Hiver P., Al-Hoorie A.H., & Mercer S. (2021). Student engagement in the language classroom. Multilingual Matters.

[ref23] Hu L., & Bentler P.M. (1999). Cutoff criteria for fit indexes in covariance structure analysis: Conventional criteria versus new alternatives. Structural Equation Modeling a Multidisciplinary Journal, 6(1), 1–55. 10.1080/10705519909540118

[ref24] Khajavy G.H., MacIntyre P.D., & Hariri J. (2021). A closer look at grit and language mindset as predictors of foreign language achievement. Studies in Second Language Acquisition, 43(2), 379–402. 10.1017/S0272263120000480

[ref25] Kline R.B. (2016). Principles and practice of structural equation modeling (Fourth edition). The Guilford Press.

[ref26] Kunnan A.J. (1998). An introduction to structural equation modeling for language assessment research. Language Testing, 15, 295–332. 10.1177/026553229801500302

[ref27] Larson-Hall J. (2015). A Guide to Doing Statistics in Second Language Research using SPSS and R. Routledge.

[ref28] Li M. (2024). The interplay between perceived teacher support, grit, and academic engagement among Chinese EFL learners. Learning and Motivation, 85, 101965. 10.1016/j.lmot.2024.101965

[ref29] Mercer S., & Dörnyei Z. (2020). Engaging language learners in contemporary classrooms: Strategies for fostering rapport and motivation. Cambridge University Press.

[ref30] Mystkowska-Wiertelak A. (2020). Teachers’ accounts of learners’ engagement and disaffection in the language classroom. The Language Learning Journal, 50(3), 1–13. 10.1080/09571736.2020.1800067

[ref31] Namaziandost E., Çakmak F., Heydarnejad T., & Rezai A. (2024). The predictive effects of learner autonomy and academic engagement on willingness to communicate, foreign language learning self-esteem, and L2 grit in an EFL context. Acta Psychologica, 250, 104528. 10.1016/j.actpsy.2024.104528 39405746

[ref32] Pourgharib B., & Shakki F. (2024). The interplay between English teachers’ rapport and immediacy and the students’ academic motivation. Learning and Motivation, 87, 101991. 10.1016/j.lmot.2024.101991

[ref33] Schaufeli W.B., Martínez I.M., Pinto A.M., Salanova M., & Bakker A.B. (2002). Burnout and engagement in university students. Journal of Cross-Cultural Psychology, 33(5), 464–481. 10.1177/0022022102033005003

[ref34] Shakki F. (2022). Iranian EFL students’ L2 engagement: The effects of teacher-student rapport and teacher support. Language Related Research,13(3), 175–198. 10.52547/LRR.13.3.8

[ref35] Solhi M., Derakhshan A., & Ünsal B. (2023). Associations between EFL students’ L2 grit, boredom coping strategies, and regulation strategies: A structural equation modeling approach. Journal of Multilingual and Multicultural Development, 46(2), 224–243 10.1080/01434632.2023.2175834

[ref36] Sun P.P., Zhang J., & Zhao X. (2024). Modeling speaking performance in young learners of Chinese as a heritage language: The interplay of L2 grit, motivational intensity, and willingness to communicate. System, 126, 103490. 10.1016/j.system.2024.103490

[ref37] Teimouri Y., Plonsky L., & Tabandeh F. (2022). L2 grit: Passion and perseverance for second-language learning. Language Teaching Research, 26(5), 893–918. 10.1177/1362168820921895

[ref38] Van Uden J.M., Ritzen H., & Pieters J.M. (2014). Engaging students: The role of teacher beliefs and interpersonal teacher behavior in fostering student engagement in vocational education. Teaching and Teacher Education, 37, 21–32. 10.1016/j.tate.2013.08.005

[ref39] Wang Y.L., Derakhshan A., & Zhang L. J. (2021). Researching and practicing positive psychology in second/foreign language learning and teaching: The past, current status and future directions. Frontiers in Psychology, 12, 1–10. 10.3389/fpsyg.2021.731721 PMC8417049 34489835

[ref40] Wilson J.H., Ryan R.G., & Pugh J.L. (2010). Professor–Student Rapport scale predicts student outcomes. Teaching of Psychology, 37(4), 246–251. 10.1080/00986283.2010.510976

[ref41] Yuan L. (2024). EFL teacher-student interaction, teacher immediacy, and Students’ academic engagement in the Chinese higher learning context. Acta Psychologica [Psychological Act], 244, 104185. 10.1016/j.actpsy.2024.104185 38364636

[ref42] Zhang J. (2023). Modelling the interplay of writing achievement goals and grit in predicting L2 writing achievements. System, 117, 103118. 10.1016/j.system.2023.103118

[ref43] Zhang J., & Zhang L.J. (2023). Examining the relationship between English as a foreign language learners’ cognitive ability and L2 grit in predicting their writing performance. Learning and Instruction, 88, 101808. 10.1016/j.learninstruc.2023.101808

[ref44] Zhi R., & Wang Y. (2024). On the relationship between EFL students’ attitudes toward artificial intelligence, teachers’ immediacy and teacher-student rapport, and their willingness to communicate. System, 124, 103341. 10.1016/j.system.2024.103341

